# Latent profile analysis of accelerometer-measured sleep, physical activity, and sedentary time and differences in health characteristics in adult women

**DOI:** 10.1371/journal.pone.0218595

**Published:** 2019-06-27

**Authors:** Kelsie M. Full, Kevin Moran, Jordan Carlson, Suneeta Godbole, Loki Natarajan, Aaron Hipp, Karen Glanz, Jonathan Mitchell, Francine Laden, Peter James, Jacqueline Kerr

**Affiliations:** 1 Division of Epidemiology and Community Health, University of Minnesota, Minneapolis, MN, United States of America; 2 Department of Family Medicine & Public Health, University of California San Diego, La Jolla, California, United States of America; 3 Department of Preventive Medicine, Northwestern University Feinberg School of Medicine, Chicago, Illinois, United States of America; 4 Center for Children’s Healthy Lifestyles and Nutrition, Children's Mercy Hospital, Kansas City, Missouri, United States of America; 5 Department of Parks, Recreation, and Tourism Management, North Carolina State University, Raleigh, North Carolina, United States of America; 6 Perelman School of Medicine and School of Nursing, University of Pennsylvania, Philadelphia, Pennsylvania, United States of America; 7 Division of Gastroenterology, Hepatology and Nutrition, Children’s Hospital of Philadelphia, Philadelphia, Pennsylvania, United States of America; 8 Departments of Environmental Health and Epidemiology, Harvard T.H. Chan School of Public Health, Harvard University, Boston, Massachusetts, United States of America; 9 Channing Division of Network Medicine, Brigham and Women’s Hospital and Harvard Medical School, Harvard University, Boston, Massachusetts, United States of America; Pennington Biomedical Research Center, UNITED STATES

## Abstract

**Objectives:**

Independently, physical activity (PA), sedentary behavior (SB), and sleep are related to the development and progression of chronic diseases. Less is known about how rest-activity behaviors cluster within individuals and how rest-activity behavior profiles relate to health. In this study we aimed to investigate if adult women cluster into profiles based on how they accumulate rest-activity behavior (including accelerometer-measured PA, SB, and sleep), and if participant characteristics and health outcomes differ by profile membership.

**Methods:**

A convenience sample of 372 women (mean age 55.38 + 10.16) were recruited from four US cities. Participants wore ActiGraph GT3X+ accelerometers on the hip and wrist for a week. Total daily minutes in moderate-to-vigorous PA (MVPA) and percentage of wear-time spent in SB was estimated from the hip device. Total sleep time (hours/minutes) and sleep efficiency (% of in bed time asleep) were estimated from the wrist device. Latent profile analysis (LPA) was performed to identify clusters of participants based on accumulation of the four rest-activity variables. Adjusted ANOVAs were conducted to explore differences in demographic characteristics and health outcomes across profiles.

**Results:**

Rest-activity variables clustered to form five behavior profiles: Moderately Active Poor Sleepers (7%), Highly Actives (9%), Inactives (41%), Moderately Actives (28%), and Actives (15%). The Moderately Active Poor Sleepers (profile 1) had the lowest proportion of whites (35% vs 78–91%, p < .001) and college graduates (28% vs 68–90%, p = .004). Health outcomes did not vary significantly across all rest-activity profiles.

**Conclusions:**

In this sample, women clustered within daily rest-activity behavior profiles. Identifying 24-hour behavior profiles can inform intervention population targets and innovative behavioral goals of multiple health behavior interventions.

## Introduction

Low levels of physical activity (PA) and high levels of sedentary behavior (SB) have been identified as major modifiable risk factors for chronic diseases such as diabetes, obesity, and cardiovascular disease [1–3]. Similarly, sleep is increasingly seen as an important determinant of overall health, with evidence suggesting a link between total sleep time and all-cause mortality and the development of chronic diseases [4–6].

Previous research has primarily examined these rest-activity behaviors (PA, SB, and sleep) individually and their associations with relevant health outcomes (variable-centered analyses). In reality, these rest-activity behaviors do not occur in isolation, and time spent in PA, SB, and sleep aggregates to make up the finite 24-hour day. Variable-centered analyses do not allow for us to develop an understanding of how rest-activity behaviors aggregate within individuals to impact their health. Further, evidence indicates that daily rest-activity activity behaviors are interrelated, suggesting that an individual’s engagement in one activity is associated with engagement in another. In an NHANES sample of 2,989 adults, lower levels of daily PA was associated with higher self-reported sleep disturbance scores [7]. Little research has been conducted to move beyond studies focused on one or two daily activities, to explore how 24-hour rest-activity behaviors cluster within individuals to form patterns in 24-hour behavior (person-centered analyses) [8].

The clustering analysis (person-centered analyses) approach has been widely used to explore daily activity behavior profiles in youth and adolescent samples and their associations to health outcomes [9–12]. In an analysis of 5710 children from 12 countries, Dumuid et al. found that distinct cluster profiles in health behaviors emerged globally for children and that cluster membership was associated with differences in BMI for both boys and girls. The children clustered in the “sitters” profile were more likely to be obese/overweight than those in the other profile groups [9]. Latent Profile Analysis, one clustering analysis approach, allows for the identification of naturally occurring subsets of individuals based on how their rest-activity behaviors cluster and allows for the exploration of differences between these behavior profiles. Despite its common use in adolescent and child samples, the clustering approach has not yet been used extensively to examine rest-activity behavior profiles in adult samples. Further, in the few existing studies applying clustering analyses in adult samples, results have been limited by the use of self-reported measures of PA, SB, and sleep [13] which may bias clustering analyses, as individuals will tend to have similar reporting biases across behaviors [14]. There is a need for more studies using objective measures of rest-activity behaviors to examine 24-hour behavior profiles in adults. Identifying 24-hour behavior profiles in adults and differences in demographic characteristics across profiles could inform behavior change intervention targets and target population groups in greatest need of lifestyle activity interventions.

This study aimed to leverage a free-living cohort of middle aged to older women across the US, including working adults, nurses, and breast cancer survivors. Using objectively measured rest-activity behavior data we will apply clustering analyses to explore: 1) whether women cluster into rest-activity behavior profiles based on how they accumulate total sleep time, sleep efficiency, PA, and SB, and if 2) if demographic characteristics and health outcomes vary according to rest-activity behavior profiles.

## Methods

### Sample

Our convenience sample included 372 adult women participating in studies across four university sites in the US (University of California, San Diego (UCSD), Washington University in St. Louis (WUSTL), Harvard University (Harvard), and University of Pennsylvania (UPenn)) involved in the NCI-funded Transdisciplinary Research in Energetics and Cancer (TREC) initiative [15]. Across institutions, participants were recruited from existing studies and/or potential study participant rosters. For this cross-sectional analysis, data were obtained from women participating in cross-sectional observation studies at Harvard, UPenn and WUSTL and a UCSD weight loss intervention. All data were collected at baseline and therefore there was no need to account for intervention effect. The resulting aggregate sample includes working adults living in San Diego, CA (UCSD; N = 73) and Saint Louis, MO (WUSTL; N = 78), nurses throughout the US participating in the Nurses’ Health Study II (Harvard; N = 93), and breast cancer survivors living in Philadelphia, PA (UPenn; N = 128). For the UCSD study, participants initially screened as ineligible for the intervention study were contacted for the current study and if interested were included in analyses. To be included in the present study, all participants completed a new consent form and additional study measures following a standard protocol. At all sites, women had to meet the following eligibility criteria: be between 21–75 years old, have a self-reported BMI between 21–39.9 kg/m^2^, have the ability to ambulate unassisted, not be pregnant or breast-feeding, and be willing to wear monitoring devices for 7 days. Site-specific eligibility criteria included: current full or part-time employment (WUSTL) and a previous breast cancer diagnosis (UPenn). The Harvard sample was selected to evenly represent all Census regions of the US (African American were oversampled).

Data collection was conducted remotely for the Penn, Harvard, and WUSTL sites and in person for the UCSD site. In the remote data collection scenarios, accelerometers were mailed to 87% of participants with instructions, a link to an instructional video, and a paper survey. Paper surveys were completed by participants and returned via mail. For the remaining participants at UCSD, accelerometers were received and returned in person. Study protocols specific to the collection of accelerometer data and survey completion were identical across all sites and all data used in these analyses were centrally pooled and uniformly processed at UCSD [16]. The accelerometer protocol included providing participants with reminder cards to place by their bed and a paper log to track device wear days. Additionally, reminder calls were made to the participant twice over the course of their wear period. The institutional review boards at the University of California, San Diego, Washington University in St. Louis, Harvard University, and University of Pennsylvania approved the study protocol and consent forms.

### Measures and data processing

#### Physical activity & sedentary behavior

Moderate-to-vigorous physical activity (MVPA) and sedentary behavior were measured objectively using a hip-worn Actigraph GT3X+ (ActiGraph, LLC; Pensacola, FL), a lightweight triaxial accelerometer. The ActiGraph device has been validated and calibrated for physical activity and sedentary behavior measurement in the field and in controlled conditions [17]. The accelerometer measures duration, frequency and intensity of activity. Participants were asked to wear the device during waking hours for 7 consecutive days. In order for a day of accelerometer data to be considered a valid wear day, the participant had to wear the device for at least 10 hours. Participants with fewer than five valid days of wear time were not included in analyses. Non-wear time was defined as 90 consecutive minutes of zero counts and was removed from all analyses [18]. The following cut-points were used to calculate daily times in each activity intensity level: sedentary 0–99 counts/min; MVPA ≥ 2020 counts/min [19]. All other counts were categorized as light activity and not used in the present analysis. To adjust for the relationship between daily wear-time and sedentary behavior time, sedentary behavior was calculated as a percentage of wear-time. Daily levels of MVPA minutes and percent sedentary time were aggregated to compute an average daily value and a standard deviation (SD) of these daily values.

#### Sleep

Sleep was measured using a wrist-worn Actigraph GT3X+ accelerometer, in conjunction with a self-reported sleep log. Participants were instructed to wear the wrist accelerometer for 24 hours per day during the monitoring period, and to record their in-bed and out-of-bed times for each sleep period in the sleep log. Sleep was scored using the log-reported in bed and out of bed time to define the major sleep period, adhering to SBSM actigraphy use guidelines [20]. A validated algorithm was used to categorize each minute of the identified sleep period as either “awake” or “asleep” [21]. This algorithm has been previously validated for use in GT3x accelerometer devices [22]. Total sleep time was derived as the total number of minutes categorized as “sleep” during each sleep period. Sleep efficiency was defined as the percentage of the in-bed period that was categorized asleep. Hip and wrist accelerometers were worn on concurrent days. Nightly levels of sleep time and sleep efficiency were aggregated to compute an average nightly value.

#### Self-report measures

All participants completed a study survey when they received their measurement devices. The survey assessed age (years), race/ethnicity, marital status, education, self-rated health, and disease diagnoses. Race/ethnicity was dichotomized into white or racial/ethnicity minority (including Hispanic/Latino, Asian, Black or African American, American Indian or Alaska Native, Native Hawaiian or Pacific Islander, or Other). Marital status was dichotomized into married (or living with a partner) or non-married (never married, divorced or separated, or widowed). Education was dichotomized into having a college degree (bachelor or graduate degree) or not (grade school or some high school, high school diploma or G.E.D., or some college or Associate Degree). BMI (kg/m^2^) was calculated using self-reported height and weight. Self-rated health was rated on a 1 (“Poor”) to 5 (“Excellent”) scale. Health conditions were assessed via the question “Do you currently have, or has a physician told you that you have any of the following health conditions?” A count of health conditions was created from a list of 16 possibilities, and participants were grouped categorically as: 0, 1, 2, or ≥ 3. Physical functioning was measured using a subset of the Short Form-36 Health Survey [23]. Participants were asked about the extent to which their health limited in daily activities including but not limited to: vigorous exercise, carrying groceries, climbing multiple flights of stairs, walking several blocks, and bathing one’s self, etc. Scores are converted to a 0–100 scale, with a higher score indicating better physical functioning.

### Statistical analyses

Analyses were conducted at the participant level. The four rest-activity behaviors of interest (nightly sleep time, nightly sleep efficiency, MVPA min/day, and percent time spent sedentary) were assessed at the daily level. Two participant-level variables were created for each behavior by taking the mean and standard deviation (SD) of each daily-level variable across days. Bivariate Pearson’s correlations were used to investigate associations among the four daily mean variables.

Latent profile analyses were conducted on the four participant-level daily mean variables to derive mutually exclusive classes (clusters) that maximize between-group variance while minimizing within-group variance. The model uses the distributional assumptions to find classes. This approach was chosen over a regression model approach, with the intention of going beyond examining if two of the four rest-activity behavior variables are related to one another to examine if multiple rest-activity behaviors cluster together within individuals. This approach has the potential to generate participant behavior profiles that will allow for the further exploration of differences in demographics and health outcomes across behavior profile groups.

Model fits were compared to derive the number of profiles that best fit the data [24], using the Akaike information criterion (AIC) statistic for model fit, a Lo-Mendell-Rubin adjusted likelihood ratio test (LMRT) [25], the Bayesian information criteria (BIC) which assesses the relative quality of the model in each successive iteration [26], as well as the sample size adjusted BIC and the interpretability of the profiles. Due to the large proportion of participants in one profile from the latent profile analysis, a second latent profile analysis was conducted on the participants in this large profile. Resulting behaviour profiles were labelled according to meeting or not meeting the 2018 national PA guidelines [27] and the National Sleep Foundation sleep duration recommendations [28].

Once the continuous rest-activity behavior profiles were identified, we investigated whether we could identify differences in social demographic characteristics across the profile groups. Analysis of variance (ANOVA) with Bonferroni post-hoc tests were used to compare differences in age, race/ethnicity, and education across the latent profiles (separate models). To account for the differences in sample populations, these models adjusted for study site and additional demographic characteristics (i.e. the model comparing age adjusted for race/ethnicity and education). Additional ANOVA models were then used to assess differences in participant health characteristics previously associated in the literature with daily rest-activity variables (including: BMI, self-rated health, physical functioning, and number of health conditions) across behavior profiles. Health conditions were grouped categorically as: 0, 1, 2, or ≥3. Models adjusted for covariates selected a priori including: age, race/ethnicity, education, and site.

MPlus 6.12 was used for all of the statistical analyses.

## Results

Of the 402 people recruited to the study, 368 (92%) returned both wrist and hip accelerometers with valid wear, and an additional 4 people returned accelerometers with valid data after requesting a re-wear, resulting in a final analytic sample of 372 participants. The all-female study sample had an average age of 55.38 (SD = 10.16) years (Table 1). Participants were mostly white (78%), married (71%), employed (74%), and college educated (68%). All of the participants from University of Pennsylvania were breast cancer survivors (n = 128, 34%). Participants achieved an average of 21.14 (SD = 18.92) minutes of daily MVPA, while 62% percent of their daily time was spent being sedentary. The mean nightly sleep time was 408.88 (SD = 55.74) minutes or 6 hours and 49 minutes. On average, participants’ estimated sleep efficiency was 86%.

**Table 1:** Demographic characteristics of sample (N = 372).

	Mean [SD] N (%)
**Age**	55.38 [10.16]
**Race/Ethnicity**	
White non-Hispanic	285 (77)
All other	80 (22)
**Marital Status**	
Married	259 (70)
Not Married	106 (29)
**Employment**	
Not Employed	94 (25)
Employed	266 (72)
**Education**	
Below college	117 (31)
College and above	248 (67)
**Daily wear time (min)**	872.29 [76.64]
**MVPA per day (min)**	21.14 [18.92]
**Percent wear time sedentary (%)**	62 [9]
**Nightly sleep time (min)**	408.88 [55.74]
**Sleep efficiency**	85.56 [7.46]
**Health Characteristics**	
BMI	27.79 [6.51]
Physical Functioning (0–100)	87.58 [17.88]
Self-Rated Health (1–5)	3.54 [0.91]
Health Conditions (0, 1, 2, 3+)	1.55 [1.54]
Health Conditions Excluding Sleep Problems (0, 1, 2, 3+)	1.48 [1.47]

Univariate Pearson’s correlations were run to examine possible correlations between the rest-activity behavior variables. As expected, percent sedentary time (SB) and daily MVPA showed a moderate inverse correlation (*r* = -0.31, p < 0.01). Of note, nightly sleep time and sleep efficiency were only moderately correlated (*r* = 0.47, p < 0.01). Neither MVPA nor SB were significantly correlated with either sleep behavior variable.

The model fit indices for the latent profile analyses are available in Table 2. The reduction in the AIC and BIC supports a 3, 4 or 5-class solution over the 6-class solution. When all of the indices and the LMRT (p < 0.001) were considered, the 3-class solution appeared to be the overall best fit. Latent class probabilities for each class were 7% (n = 27) in class 1, 9% (n = 34) in class 2, and 80% (n = 312) in class 3. We conducted an additional LPA of the largest profile which captured 80% of participants. Similar to the first LPA, the model fit indices and interpretation of the data supported the 3-class solution fit. Respective to the entire sample, latent class probabilities for these classes were 41% (n = 151) for class 3.1, 28% (n = 105) for class 3.2, and 15% (n = 55) for class 3.3.

**Table 2:** Latent profile analysis model fit indices.

**Initial Latent Profile Analyses**				
	**AIC**	**BIC**	**sBIC**	**LMRT** **p-value**
1 Class	8950.73	8982.10	8956.72	N/A
2 Class	8789.40	8840.38	8799.14	0.009
3 Class	8691.47	8762.06	8704.95	0.009
4 Class	8664.85	8755.05	8682.08	0.861
5 Class	8624.29	8734.10	8645.26	0.098
6 Class	8647.32	8776.73	8672.03	0.533
**Secondary Latent Profile Analyses**				
3.1 Class	6842.10	6872.04	6846.67	N/A
3.2 Class	6781.05	6829.71	6788.48	< .0001
3.3 Class	6758.71	6826.09	6769.00	0.053
3.4 Class	6743.58	6829.67	6756.72	0.082

Table 3 present the behavioral characteristics for the five observed profiles. Profile 1 was labelled “Moderately Active Poor Sleepers” due to a mean daily MVPA (19.30 minutes) below the 2018 national PA guidelines of 30 minutes of daily MVPA, and a mean total sleep time (328.93 minutes /5.5 hours per night) that does not meet National Sleep Foundation sleep duration recommendations (7–9 hours of sleep per night). Profile 2 was labelled “Highly Actives” due to high levels of MVPA (65.37 minutes) that exceed 2018 PA guidelines and a total sleep time just below sleep duration recommendations (406.70 minutes /6.78 hours per night). Profile 3.1 was labelled “Inactives” due to a very low mean MVPA (6.77 minutes) that does not meet PA guidelines, and a mean total sleep that is closely in line with sleep duration recommendations (415.81 minutes / 6.93 hours per night). Profile 3.2 was labelled “Moderately Actives” due to their moderate levels of MVPA (19.81 minutes), and a total sleep time that is also closely in line with sleep duration recommendations (415.96 minutes / 6.93 hours per night). Profile 3.3 was labelled “Actives” due to a mean daily MVPA (36.06 minutes) that meets PA guidelines and a total sleep time that nearly meets sleep duration recommendations (417.60 minutes / 6.96 hours per night).

**Table 3:** Observed time spent in rest-activity behavior by behavior profile [mean (SD)].

		Profile 1 Moderately Active Poor Sleepers n = 27 (7%)	Profile 2 Highly Actives n = 34 (9%)	Profile 3.1 Inactives n = 151 (41%)	Profile 3.2 Moderately Actives n = 105 (28%)	Profile 3.3 Actives n = 55 (15%)
MVPA per day (min)		19.30 (18.86)	65.37 (14.67)	6.77 (3.95)	19.81 (4.44)	36.06 (5.46)
Percent Wear-Time Sedentary (%)		64 (11)	56 (10)	66 (7)	59 (7)	62 (8)
Total Sleep Time (min)		328.93 (58.11)	406.70 (50.31)	415.81 (54.36)	415.96 (47.24)	417.60 (45.77)
Sleep Efficiency (%)		66 (7)	87 (6)	86 (5)	87 (4)	89 (5)

Table 4 presents the differences in rest-activity behavior means and SEs, demographics, and health characteristics for the five rest-activity behavior profiles. Rest-activity behavior means and health characteristics were adjusted for age, race, marital status, education, and site.

**Table 4:** Adjusted means for behaviors, demographics, and health outcomes by behavior profile and Bonferroni test results.

	Profile 1 Moderately Active Poor Sleepers n = 27 Mean (SE)	Profile 2 Highly Actives n = 34 Mean (SE)	Profile 3.1 Inactives n = 151 Mean (SE)	Profile 3.2 Moderately Actives n = 105 Mean (SE)	Profile 3.3 Actives n = 55 Mean (SE)
MVPA per day (min)	19.01 (1.61)^b^^,^^c^^,^^e^	65.06 (1.33)^τ^	7.15 (0.61)^τ^	19.73 (0.73)^b^^,^^c^^,^^e^	35.39 (1.0)^τ^
Percent Wear-Time Sedentary	64.24 (1.76)^b^	55.82 (1.45)^a^^,^^c^^,^^e^	65.45 (0.67)^b^^,^^d^	59.03 (0.79)^c^	61.98 (1.09)^b^
Nightly Sleep Time (min)	336.67 (11.04) ^τ^	402.98 (9.50)^a^	415.96 (4.29)^a^	414.93 (5.13)^a^	416.78 (7.18)^a^
Sleep Efficiency	68.17 (1.05) ^τ^	86.63 (0.91)^a^	86.35 (0.41)^a^	87.33 (0.49)^a^	88.50 (0.69)^a^
MVPA Daily SD	10.97 (1.56)^b^^,^^c^^,^^e^	32.03 (1.29)^τ^	5.96 (0.59)^τ^	14.51 (0.70)^b^^,^^c^^,^^e^	21.40 (0.97)^τ^
Percent Wear-Time Daily SD	7.60 (0.67)	7.29 (0.55)	7.60 (0.25)	7.64 (0.30)	7.89 (0.42)
Nightly Sleep Time SD	80.08 (6.20)^τ^	53.44 (5.26)^a^	61.03 (2.38)^a^	52.53 (2.84)^a^	52.70 (3.97)^a^
Nightly Sleep Efficiency SD	10.26 (0.73)^τ^	5.42 (0.62)^a^	5.38 (0.28)^a^	4.42 (0.33)^a^	4.87 (0.47)^a^
Age	53.12 (2.04)	53.79 (1.73)	57.97 (0.78)^e^	54.58 (0.95)	51.81 (1.29)^c^
Percent White	35.04 (7.76)^τ^	90.45 (6.83)^a^	77.90 (3.13)^a^	84.22 (3.73)^a^	81.59 (5.16)^a^
Percent Married	62.48 (9.44)	69.42 (80.14)	68.60 (3.66)	74.25 (4.38)	75.05 (60.25)
Percent College Grad	28.34 (9.31)^τ^	90.30 (8.01)^a^	68.32 (3.69)^a^	65.62 (4.42)^a^	76.19 (6.07)^a^
BMI kg/m2	28.89 (1.33)	26.20 (1.12)	29.15 (0.51)^d^	26.61 (0.61)^c^	26.71 (0.85)
Physical Functioning (0–100)	78.90 (3.67)^d^	90.63 (3.11)	85.01 (1.44)	91.22 (1.71)^a^	90.12 (2.34)
Self-Rated Health (1–5)	3.21 (0.18)	3.75 (0.15)	3.42 (0.07)	3.61 (0.08)	3.80 (0.11)
Number of Health Conditions (Categorical: 0, 1, 2, 3+)	1.86 (0.23)	1.13 (0.19)	1.40 (0.09)	1.33 (0.11)	1.10 (0.15)
Number of Health Conditions Excluding Sleep Problems (Categorical: 0, 1, 2, 3+)	1.80 (0.23)	1.08 (0.19)	1.34 (0.09)	1.32 (0.11)	1.05 (0.15)

^τ:^ Significant difference from all other Profiles

^a:^ Significant difference from Profile 1

^b:^ Significant difference from Profile 2

^c:^ Significant difference from Profile 3.1

^d:^ Significant difference from Profile 3.2

^e:^ Significant difference from Profile 3.3. differences all p>0.05.

All models adjusted for age, race, marital status, education, and site. Bonferroni post-hoc analyses were performed.

The rest-activity behavior profile with the largest number of participants was the Inactives profile (profile 3.1, n = 151, 41%). Women in this group were close to meeting sleep recommendations but spent more time in SB (66% time) and were the least physically active (7 minutes/day of MVPA) compared to women in the other groups.

Overall, sleep characteristics did not vary greatly across all profiles. Profile 1 (Moderately Active Poor Sleepers) was the only group to have significantly poorer sleep characteristics than the other four profiles. Additionally, the comparison of SDs of the sleep variables indicates more night-to-night variability in the sleep of women in Profile 1 compared to the other behavior profiles. Women in Profile 2 (Highly Actives) did not meet sleep duration guidelines, but had moderately high sleep efficiency (87%) and overall sleep characteristics did not differ significantly from profiles 3.1, 3.2, or 3.3.

Only 2 profiles (Profile 2 and 3.3), comprising 24% of women, met daily MVPA recommendations. Mean daily MVPA was as high as 65 minutes/day in the Highly Actives and as low as 6.77 minutes/day for the Inactives. Sedentary time did not vary across behavior profiles to the extent that MVPA did, as there was only a 10% difference in sedentary time between the profile with the most (profile 3.1: 66% sedentary) and least SB (profile 2: 56% sedentary).

In our additional exploratory analyses demographic characteristics did not differ significantly across the behavior profile groups. There were no significant differences in profile membership based on study site (data not shown). Age was largely comparable across rest-activity behavior profiles, with one significant difference (p = .001) between the Inactives (57.97 years, SD: 0.78) and the Actives (51.81 years, SD: 1.29). Race-ethnicity and education did not vary across all of the profiles, however the Inactives had a significantly lower proportion of whites (35% vs 78–91%, p < .001), and a lower proportion of college graduates (28% vs 68–90%, p = .004) than all of the other behavior profiles. Marital status did not differ significantly across profiles.

Additionally, health outcomes did not vary significantly across all rest-activity profiles. Significant differences in BMI were only observed between the Inactives, who reported the highest BMI (28.89 kg/m2, SD: 1.33), and the Moderately Actives (26.61 kg/m2, SD:0.61), but not the other profiles. Physical functioning was also largely similar among behavior profiles, with significant differences observed between the Moderately Active Poor Sleepers, who reported the lowest physical functioning scores (78.90, SD: 3.67), and the Moderately Actives who reported the highest physical functioning scores (91.22, SD: 1.71). Self-rated health and reported number of health conditions did not differ significantly across behavior profiles, however means trended in the expected directions (poorer health in the Moderately Active Poor Sleepers and the Inactives compared to better self-rated health in the Highly Actives and the Actives). Additionally, the women in the Moderately Active Poor Sleepers group were more likely to report working a regular night shift (12% vs. 0–2%) and having a sleep disorder (14% vs. 7–9%) than members of the other behavior profiles, although these differences were not statistically significant (data not presented in table).

## Discussion

This was one of the first studies to use objectively measured PA, SB, and sleep to explore the clustering of 24-hour rest-activity behaviors in adult women. Five different rest-activity behavior profiles emerged from our analysis. Demographic characteristics and health outcomes did not vary significantly across behavior profiles. However, we found the behavior profile with the least MVPA and highest percent time in SB had the highest mean BMI, and the behavior profile with the shortest nightly sleep time and lowest sleep efficiency had the poorest physical functioning. The results of this clustering analysis support future studies further exploring the interrelationship of 24-hour rest-activity behaviors among adults.

In our sample of adult women, MVPA was the largest discriminator across the resulting rest-activity behavior profiles. Overall, only 24% of women in our sample achieved mean daily minutes of MVPA that met the 2018 PA guidelines for adults, which is similar to recent population estimates of women meeting PA guidelines in the United States [27]. Additionally, time spent in SB did not vary across rest-activity behavior profiles in our analysis. If estimates from our analysis are accurate, women in the US are spending approximately 60% of their time in SB, equivalent to 14 hours a day. Both the MVPA and SB results from our analysis provide further evidence of opportunities for intervention among adult women. However, it is important to note that in our sample SB estimates were high even in the profiles with higher estimates of MVPA. Although our study is cross-sectional, these findings may explain why targeting PA does not necessarily result in decreases in SB [29] and targeting SB does not necessarily lead to significant increases in MVPA [30]. These results provide support of the numerous lines of evidence establishing the adverse health effects of SB, independent of PA (i.e., these are distinct behaviors) [31,32].

In this study, sleep characteristics did not vary significantly across the resulting 24-hour rest-activity behavior profiles. Overall, the entire sample of women had average nightly sleep times that were just under meeting the national recommendations of 7–9 hours per night [28]. Only one profile had significantly less total sleep time and lower sleep efficiency than the other four profiles. Further, there was essentially no correlation observed between MVPA and nightly sleep time in this sample. Previous studies that have documented positive associations between MVPA and sleep time have typically relied on self-report for at least one of the behaviors [33], which may explain the difference in findings. A small but positive correlation was observed between MVPA and sleep efficiency, suggesting that women with more minutes of MVPA have better sleep efficiency. The profile with the poorest sleep characteristics still achieved moderate amounts of MVPA. Therefore, experiencing poor sleep may not be related to the amount of daily MVPA achieved among women [34]. Further, the profile with the poorer sleep characteristics were more likely to be non-white. The demographic characteristics of this profile are consistent with studies indicating that racial-ethnic minorities report experiencing shorter sleep duration sand worst sleep quality than their white counterparts [35]. Considering the health implications of optimal sleep, this result may be meaningful for targeting at-risk population subgroups in need of sleep duration and sleep quality interventions [36].

Consistent with previous research, in our study the behavior profile with the lowest minutes of daily MVPA and highest SB had the highest BMI [27]. The behavior profile with the poorest physical functioning was the profile with the poorest sleep characteristics, which may have been a cause and/or consequence of the poor sleep experienced in that group. No other significant differences were observed in health characteristics across the behavior profiles, though it is possible that a larger sample size may be needed to adequately test for differences.

This study has several limitations. While the study data were collected daily allowing for possible longitudinal assessment, this study was cross-sectional and averages of daily activities were assessed to answer the research questions. Due to the cross-sectional design examining causal relationships between profile membership and health conditions was not possible. Actigraphy was used to measure PA (gold standard), however this approach is limited in its ability to capture certain PA modalities, such as swimming and cycling. Accelerometers were also used to measure sleep rather than the gold standard polysomnography. While our analysis included a unique sample of women varied in age, health status, and occupation, all participants were adult women, which limits the generalizability of these findings to US adult men. Health variables were all self-reported and were limited in scope and severity. While the sample size of 372 was large enough to discern five distinct profiles, a larger sample size would have greater statistical power to discern differences between these groups. Lastly, we acknowledge that some of the model fit statistics may support that a 2-class solution better represented the participants in the second LPA, however we believe that substantive interpretation lends support for a 3-class solution to this model.

Few studies have investigated how women cluster in rest-activity behavior profiles based on their accumulation of activity throughout the 24-hour day. This study is one of the first to explore 24-hour behavior patterns in women using objective measures of 24-hour daily activity behavior. This study demonstrates that women cluster into distinct behavior profile groups based on their daily MVPA, SB, and sleep characteristics. Prior studies have relied upon self-reported measures of these behaviors and have typically examined behaviors individually, ignoring the possible interrelationships of daily activity behaviors. This analysis supports the use of future longitudinal studies to further untangle the interrelationships of these behaviors and understand how behavior profiles may be associated with health outcomes over time. Additionally, more research is needed to determine whether these behavior profiles generalize to other population subgroups (e.g., men, youth, older adults) and racial-ethnically diverse samples. While more research is needed, this study demonstrates the importance of considering the clustering of rest-activity behaviors in intervention design and population targeting. This study provides promising evidence to support the further exploration of multiple behavior change interventions and identifying populations most in need of lifestyle intervention, including the populations with the lowest MVPA and poorest sleep characteristics [36–38].

## Abbreviations

MVPA: moderate-to-vigorous physical activity
PA: Physical Activity
SB: Sedentary Behavior
SD: standard deviation
LMRT: Lo-Mendell-Rubin adjusted likelihood ratio test
sBIC: sample size adjusted Bayesian information criteria
ANOVA: analysis of variance
LPA: latent profile analysis
